# Ultrasound evaluation of the fetal fat tissue, heart, liver and umbilical cord measurements in pregnancies complicated by gestational and type 1 diabetes mellitus: potential application in the fetal birth-weight estimation and prediction of the fetal macrosomia

**DOI:** 10.1186/s13098-021-00634-7

**Published:** 2021-02-18

**Authors:** Paweł Jan Stanirowski, Agata Majewska, Michał Lipa, Dorota Bomba-Opoń, Mirosław Wielgoś

**Affiliations:** 1 1st Department of Obstetrics and Gynecology, Medical University of Warsaw, Starynkiewicza Sq. 1/3, 02-015 Warsaw, Poland; 2 Club 35. Polish Society of Gynecologists and Obstetricians, Warsaw, Poland

**Keywords:** Fetal fat-tissue, Gestational diabetes mellitus, Macrosomia, Type 1 diabetes mellitus, Ultrasound

## Abstract

**Background:**

The aim of the study was to evaluate the ultrasound-derived measurements of the fetal soft-tissue, heart, liver and umbilical cord in pregnancies complicated by gestational (GDM) and type 1 diabetes mellitus (T1DM), and further to assess their applicability in the estimation of the fetal birth-weight and prediction of fetal macrosomia.

**Methods:**

Measurements were obtained from diet-controlled GDM (GDMG1) (n = 40), insulin-controlled GDM (GDMG2) (n = 40), T1DM (n = 24) and healthy control (n = 40) patients. The following parameters were selected for analysis: fetal sub-scapular fat mass (SSFM), abdominal fat mass (AFM), mid-thigh fat/lean mass (MTFM/MTLM) and inter-ventricular septum (IVS) thicknesses, heart and thorax circumference and area (HeC/HeA; ThC/ThA), liver length (LL), umbilical cord/vein/arteries circumference and area (UmC/UmA; UvC/UvA; UaC/UaA) together with total umbilical vessels (UveA) and Wharton's jelly area (WjA). Regression models were created in order to assess the contribution of selected parameters to fetal birth-weight (FBW) and risk of fetal macrosomia.

**Results:**

Measurements of the fetal SSFM, AFM, MTFM, MTFM/MTLM ratio, HeC, HeA, IVS, LL, UmC, UmA, UaC, UaA, UveA and WjA were significantly increased among patients with GDMG2/T1DM as compared to GDMG1 and/or control groups (p < .05). The regression analysis revealed that maternal height as well as fetal biparietal diameter, abdominal circumference (AC), AFM and LL measurements were independent predictors of the FBW (p < .05). In addition, increase in the fetal AFM, AC and femur length (FL) was associated with a significant risk of fetal macrosomia occurrence (p < .05). The equation developed for the FBW estimation [FBW(g) = − 2254,942 + 17,204 * FL (mm) + 105,531 * AC (cm) + 131,347 * AFM (mm)] provided significantly lower mean absolute percent error than standard formula in the sub-group of women with T1DM (5.7% vs 9.4%, p < .05). Moreover, new equation including AC, FL and AFM parameters yielded sensitivity of 93.8%, specificity 77.7%, positive predictive value 54.5% and negative predictive value of 97.8% in the prediction of fetal macrosomia.

**Conclusions:**

Ultrasound measurements of the fetal soft tissue, heart, liver and umbilical cord are significantly increased among women with GDM treated with insulin and T1DM. In addition to standard biometric measurements, parameters, such as AFM, may find application in the management of diabetes-complicated pregnancies.

## Background

According to the recent epidemiological data, the estimated prevalence of gestational diabetes mellitus (GDM) and type 1 diabetes mellitus (T1DM) in the population of pregnant women constitutes 5.8–12.9% and 0.16–0.24%, respectively [1–3]. During pregnancy, both types of diabetes are associated with numerous adverse obstetric and neonatal outcomes, including pre-eclampsia, pre-term delivery, congenital abnormalities, perinatal death, respiratory distress syndrome, increased risk of Cesarean section, birth injuries and fetal macrosomia [3]. The latter complication affects ca. 15–20% and 40–45% of fetuses in pregnancies with concomitant GDM and T1DM, respectively [3].

For almost five decades hypothesis proposed by Pedersen, according to which elevated maternal blood glucose leads to hyperglycemia and hyperinsulinemia in the fetal circulation, is the most commonly referred explanation for the pathogenesis of fetal macrosomia in diabetes-complicated pregnancies [4]. As a result of the anabolic effects of insulin, as well as insulin-like growth factor I (IGF-I) and leptin, excessive growth of fetal soft tissues and internal organs, such as the heart and liver occurs, as confirmed in animal models and in human studies [5–9]. Importantly, the stimulating effect of growth factors refers to the entire feto-placental unit as large amounts of insulin receptors and IGF-I were detected in the placenta and Wharton’s jelly i.e. mucoid connective tissue that surrounds the two arteries and vein of the umbilical cord [10, 11].

In studies conducted in a group of neonates of diabetic mothers, a significant increase in the thickness of the soft tissue skinfolds was observed and positive correlation between the mechanical and antenatal sonographic measurements was noted [6, 9, 12, 13]. To date, numerous studies have assessed non-standard biometric parameters of the fetus by means of conventional two-dimensional ultrasound in the general obstetric population. The most commonly evaluated parameters include the abdominal, humeral and mid-thigh soft-tissue thicknesses as well as the heart morphology and length of the liver [14–17]. Regrettably, only a few of the studies have concerned pregnancies complicated by diabetes mellitus, most of which included patients diagnosed with GDM [18–26]. In addition, due to the scarcity and ambiguity of data, still little is known about ultrasound measurements of the umbilical cord in pregnancies with concomitant GDM or pre-existing diabetes [27, 28]. Finally, in individual studies conducted in diabetic populations, an attempt was made to evaluate the applicability of the above-mentioned ultrasound measurements in the estimation of the fetal birth-weight (FBW) and prediction of fetal macrosomia [18, 23, 24, 29, 30].

Considering all of the above, the aim of the present study was to evaluate ultrasound-derived measurements of the fetal soft-tissue, heart, liver and umbilical cord in pregnancies complicated by GDM/T1DM, and further to assess their efficacy in the estimation of the FBW and prediction of fetal macrosomia.

## Methods

### Patients

The study population comprised of 144 women, who delivered in the 1st Department of Obstetrics and Gynecology at the Medical University of Warsaw between October 2019 and June 2020. The Local Ethics Committee approved the study, and all participants signed their written informed consent (KB/150/2013). The inclusion criteria were as follows: maternal age > 18 years, singleton pregnancy, and gestational age > 37 weeks. Fetal malformations, two-vessel umbilical cord, intrauterine fetal growth restriction, maternal chronic or pregnancy-induced hypertension, chronic renal or hepatic disease, in vitro fertilization, preterm rupture of membranes, oligohydramnios and smoking constituted the exclusion criteria.

The participants were divided into four groups: (1) GDMG1—40 patients diagnosed with GDM and treated exclusively with diet; (2) GDMG2—40 patients diagnosed with GDM, who required additional therapy with insulin; (3) T1DM—24 patients with type 1 diabetes mellitus (all class B or C according to White classification) and (4) a control group—40 women in uncomplicated pregnancy. GDM was diagnosed based on the 75 g Oral Glucose Tolerance Test (OGTT), performed between gestational weeks 24 and 28, in accordance with the criteria defined by the World Health Organization [31]. GDM patients received dietary and physical activity advice at the initial stage, and insulin therapy was introduced only in the case of repeatedly inadequate glycemic control (fasting blood glucose level > 90 mg/dl and/or 1-h postprandial blood glucose level > 140 mg/dl) [32]. No oral hypoglycemic medications were administered to patients with GDM. T1DM patients received insulin over the entire course of pregnancy.

All study participants were followed up at the hospital ambulatory from the beginning of pregnancy. Routinely, prior to 36 weeks of gestation, visits were held once a month, and only in cases of suboptimal glycemic control, their frequency was increased to 2-week intervals. After 36 weeks, visits were held weekly until the delivery. In order to assess glycemic control in the 3rd trimester of pregnancy in all patients with GDM/T1DM, the concentration of glycosylated hemoglobin (HbA1c) was analyzed in blood prior to delivery by means of immunoturbidimetric assay (VITROS 5600, Ortho Clinical Diagnostics, USA, coefficient of variation < 2%, normal range: ≤ 6%). The physicians responsible for the recruitment and follow-up of the patients (AM and DBO) were not involved in the performance of ultrasound examinations. At the same time, both study sonographers (PJS and ML) were not informed about the patient’s diabetic status.

### Ultrasonography

A fetal ultrasound was performed in all of the study participants within the 72 h period prior to the vaginal/cesarean delivery, using a Voluson E6 ultrasound device (GE Healthcare, Chicago, USA) equipped with 1–5 MHz convex transducer by two experienced operators (PJS and ML). During each examination, the standard and non-standard biometric parameters of the fetus were assessed, including biparietal diameter (BPD), head circumference (HC), abdominal circumference (AC), femur length (FL), sub-scapular fat mass (SSFM), abdominal fat mass (AFM), mid-thigh fat mass (MTFM), mid-thigh lean mass (MTLM), heart circumference and area (HeC/HeA), thorax circumference and area (ThC/ThA), inter-ventricular septum thickness (IVS), and liver length (LL). The following parameters of the umbilical cord were also measured: circumference and area (UmC/UmA), umbilical vein circumference and area (UvC/UvA), umbilical arteries circumference and area (UaC/UaA), total umbilical vessels area (UveA), and Wharton's jelly area (WjA).

**Fig. 1:**
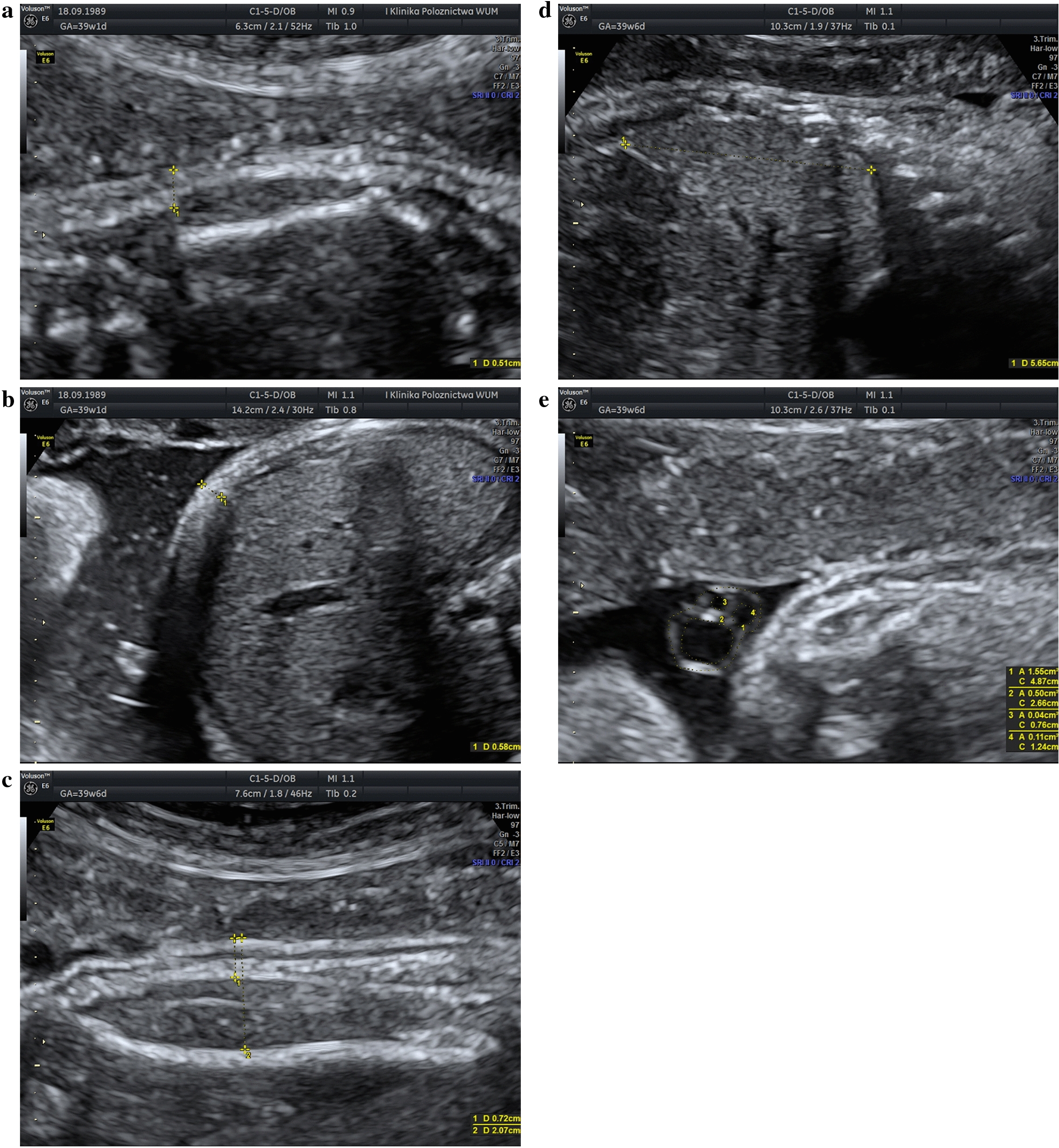
Ultrasound images presenting fetal sub-scapular fat mass (**a**), abdominal fat mass (**b**), mid-thigh fat mass/mid-thigh lean mass (**c**), liver length (**d**), umbilical cord/umbilical vein/umbilical artery circumference and area (**e**) measurement technique

For the SSFM measurement, the sagittal section of the fetal trunk was obtained, visualizing the entire scapula, with one caliper placed on the skin surface and the second caliper placed at the level of the subcutaneous tissue, perpendicularly to the bone at its distal end (Fig. 1a) [19, 33]. The AFM was determined by measuring the thickness of the anterior abdominal subcutaneous tissue. A transverse section of the fetal trunk at the level of the umbilical cord was obtained, with the fetal abdomen free from contact with extremities and with the amniotic fluid between the fetus and the uterine wall. The first caliper was placed between the amniotic fluid and the fetal skin, and the second one between the subcutaneous fat tissue and the fetal liver (Fig. 1b) [19, 33]. The MTFM and MTLM parameters were evaluated in the standard longitudinal section used for femur length measurement, in the middle of the fetal thigh [16]. For both MTFM and MTLM measurements the first caliper was placed on the skin surface, and the second caliper was placed at the level of the subcutaneous tissue (MTFM), or at the outer margin of the femur (MTLM) (Fig. 1c). The HeC/HeA and ThC/ThA measurements were performed after a good four-chamber view with complete ribs on both sides of the thorax was obtained, during heart diastole, and using the ellipse method, as described by Awadh et al. [34]. The IVS thickness was measured halfway between the apex and the crux of the heart, during maximum ventricular filling, with the septum positioned horizontally [18]. To determine the LL, the sagittal plane of the fetal abdomen was visualized and the diameter between the right hemidiaphragm dome and the tip of the right lobe was measured (Fig. 1d) [17, 21, 22]. For evaluation of the umbilical cord parameters, a cross-sectional view of a free loop was obtained and measurements of the UmC/UmA, UvC/UvA and UaC/UaA were performed using the software of the ultrasound device, as previously described (Fig. 1e) [28, 35]. The sum of three vessel areas constituted the UveA, and WjA was calculated by subtracting the UveA from the UmA. For statistical comparisons, the mean values of the UaC/UaA were calculated for each patient (mUaC/mUaA).

For the first 50 patients enrolled, reproducibility was tested and each of the parameters was measured in duplicate by two different operators (PJS and ML), blinded to each other's recordings. Precision was assessed as the coefficient of variation, and the intra- and interobserver variabilities for each measured parameter are presented in Table 1.

**Table 1:** Variability of ultrasound measurements of the selected fetal biometry parameters

Parameter	Coefficient of variation (%)	
	Intraobserver	Interobserver
SSFM	4.7	6.8
AFM	4.5	6.5
MTFM	6.4	7.8
MTLM	3.4	4.5
IVS	6.2	7.4
HeC	3.0	4.2
ThC	2.4	3.5
LL	3.5	5.4
UmC	3.6	8.1
UvC	6.6	9.9
mUaC	11.1	13.4

*SSFM* sub-scapular fat mass, *AFM* abdominal fat mass, *MTFM* mid-thigh fat mass, *MTLM* mid-thigh lean mass, *IVS* inter-ventricular septum thickness, *HeC* heart circumference, *ThC* thorax circumference, *LL* liver length, *UmC* umbilical cord circumference, *UvC* umbilical vein circumference, *mUaC* mean umbilical artery circumference

### Statistical analysis

All statistical analyses were performed using the R package v.3.6.0 (The R Foundation for Statistical Computing, Vienna, Austria). Continuous variables were compared using the Kruskal–Wallis rank sum test with the post-hoc Dunn's test, and for categorical variables the Chi-square test with the Bonferroni correction or Fisher's exact test were applied. The results were expressed as median and interquartile range [IQR], or as frequency (%).

For the purposes of correlation analysis, the following parameters were selected: maternal and gestational age, parity, maternal pre-pregnancy weight and body mass index (BMI), gestational weight gain, maternal height, glucose concentrations during OGTT, 3rd trimester HbA1c concentration, as well as ultrasound measurements of the fetal biometry, soft-tissue, heart, liver and umbilical cord. The association between FBW and selected maternal–fetal parameters was assessed with the use of Spearman's rank or Pearson's correlation coefficient (r).

Multivariate linear and logistic regression models were constructed in order to analyze independent predictors contributing to the FBW, and the risk of fetal macrosomia > 4000 g, respectively. Explanatory variables were selected from the collection of parameters included in the correlation analysis, which were subsequently discarded using a backward elimination process to maximize the value of R^2^. In addition to the above-mentioned variables the type of diabetes and fetal sex were included as possible predictors in both regression analyses.

The performances of the variables selected in the logistic model in the prediction of fetal macrosomia were evaluated using the receiver-operating characteristic (ROC) curve analysis. For each variable, the area under the curve (AUC) was calculated, and diagnostic efficiency of the established cut-off values was evaluated for sensitivity, specificity, positive predictive value (PPV) and negative predictive value (NPV). Linear regression with the FBW as the dependent variable and selected fetal measurements as independent parameters was used to derive a new best-fit formula for the antenatal birth-weight estimation. Mean absolute percent error for the new and the Hadlock formulas was calculated, and the differences were assessed by the paired *t test* [36]. A p-value of < .05 was considered statistically significant.

## Results

The characteristics of study population are shown in Table 2. Analysis revealed that gestational age was significantly lower in patients with T1DM-complicated pregnancy as compared to other groups (p < .001). Both the pre-pregnancy weight and BMI were significantly increased (p < .05), whereas gestational weight gain was significantly lower (p < .001) among patients with GDM compared to patients with pre-existing diabetes and normoglycemic pregnancy. Fasting as well as 1-h and 2-h plasma glucose concentrations during OGTT were significantly higher in both GDM groups than in the control group (p < .001). In addition, a significant increase in the HbA1c concentration was observed among women with GDMG2/T1DM compared to patients with GDM treated solely with diet (p < .05). Fetal birth-weight was significantly increased in T1DM-complicated pregnancies as compared to other groups (p < .01). Similarly, the percentage of macrosomic fetuses weighing > 4000 g was highest in the group of patients with pre-existing diabetes (p < .05). In the total study population, the percentage of macrosomic fetuses amounted to 22.2% (32/144).

**Table 2:** Clinical data and fetal ultrasound measurements in diabetic and control populations

	GDMG1 (n = 40)	GDMG2 (n = 40)	T1DM (n = 24)	Control (n = 40)	*p* value
Age (years)	32 [28–36.2]	33 [29.7–37]	33 [29–36]	30 [27.7–32]	.08
Gestational age (weeks)	39 [38–39]	39 [38–39]	38 [37–38]	39 [39–40]	< .001^c^
Gravidity	2 [1–3]	2 [1–3]	1 [1–2]	2 [1–2]	.06
Parity	2 [1–2]	2 [1–2]	1 [1–2]	2 [1–2]	.19
Pre-pregnancy weight (kg)	69.5 [61.5–75]	79 [63–89.7]	64 [55.7–70]	63.5 [56–70.5]	< .01^d^
Gestational weight gain (kg)	10 [8.5–14.2]	9 [7–13]	17 [13.7–19.5]	14.5 [11.7–18]	< .001^d,e^
Height (m)	1.65 [1.6–1.7]	1.68 [1.63–1.7]	1.64 [1.62–1.7]	1.65 [1.64–1.7]	.62
Pre-pregnancy BMI (kg/m^2^)	25.8 [23–28.3]	29.4 [22.6–33.7]	22.9 [20.4–25.4]	22.6 [21.1–25.4]	< .01^d^ < .05^e^
Fasting plasma glucose (mg/dl)^a^	90 [81.7–95]	97 [94–99]	–	80.5 [74–83.2]	< .001^f,g^
1-h plasma glucose (mg/dl)^a^	182 [158.2–190.2]	177.5 [151.7–196.7]	–	119 [105.7–142.5]	< .001^f^
2-h plasma glucose (mg/dl)^a^	153 [135.7–162]	136 [118–160]	–	102 [88.5–115.5]	< .001^f^
3rd trimester HbA1c (%)	5.1 [5–5.4]	5.7 [5.2–6.1]	5.9 [5.7–6.4]	–	< .001^h^ < .05^g^
Fetal sex					
Male	21 (52.5%)	16 (40.0%)	13 (54.2%)	25 (62.5%)	.25
Female	19 (47.5%)	24 (60.0%)	11 (45.8%)	15 (37.5%)	
Fetal birth-weight (g)	3425 [3237.5–3776.2]	3535 [3222.5–3748.7]	3945 [3600–4162.5]	3480 [3232.5–3832.5]	< .01^c^^,^^f,g^
Fetal macrosomia^b^	7 (17.5%)	7 (17.5%)	11 (45.8%)	7 (17.5%)	< .05^c^
BPD (cm)	9.27 [9–9.56]	9.29 [9.1–9.47]	8.9 [8.71–9.33]	9.28 [9.14–9.53]	< .05^c^
HC (cm)	33.4 [32.8–34.4]	33.2 [32.7–34]	32.6 [31.5–33.7]	33.8 [33.1–34.3]	< .05^h,i^
AC (cm)	34.2 [33.4–35.6]	34.8 [33.2–36]	35.4 [33.6–36.2]	34.9 [34.1–36.6]	.27
FL (cm)	7.42 [7.06–7.72]	7.51 [7.29–7.71]	7.38 [7.13–7.77]	7.47 [7.25–7.7]	.68
SSFM (cm)	0.55 [0.48–0.58]	0.62 [0.54–0.68]	0.66 [0.62–0.73]	0.53 [0.49–0.58]	< .01^c^^,j^
AFM (cm)	0.62 [0.59–0.68]	0.71 [0.65–0.76]	0.78 [0.72–0.84]	0.62 [0.56–0.7]	< .01^c^^,j^
MTFM (cm)	0.49 [0.46–0.58]	0.56 [0.47–0.65]	0.58 [0.5–0.65]	0.51 [0.45–0.56]	< .05^h,i,j^
MTLM (cm)	1.48 [1.26–1.67]	1.54 [1.32–1.74]	1.55 [1.36–1.85]	1.5 [1.31–1.58]	.45
MTFM/MTLM ratio	0.37 [0.32–0.4]	0.38 [0.34–0.41]	0.38 [0.34–0.4]	0.35 [0.31–0.38]	< .05^i,k^
HeC (cm)	13.7 [12.8–14.2]	14 [13.6–15]	14.7 [13.8–15.3]	13.9 [13.1–14.8]	< .05^h^
HeA (cm^2^)	14.5 [12.9–15.8]	15.7 [14.7–17.3]	17.2 [14.8–18.6]	14.9 [13.7–17.1]	< .05^h^
ThC (cm)	29.5 [27.7–31.4]	30.7 [29.5–32.2]	31 [30.2–31.6]	30.8 [29.9–31.9]	.07
ThA (cm^2^)	69 [61–78.3]	74.1 [68.6–82.3]	76.3 [72.8–78.4]	75 [70.7–80.4]	.07
HeC/ThC ratio	0.47 [0.44–0.47]	0.46 [0.44–0.47]	0.47 [0.44–0.49]	0.46 [0.43–0.47]	.59
HeA/ThA ratio	0.21 [0.2–0.22]	0.21 [0.19–0.22]	0.22 [0.2–0.24]	0.21 [0.19–0.22]	.60
IVS (cm)	0.36 [0.33–0.39]	0.41 [0.39–0.46]	0.47 [0.43–0.49]	0.33 [0.31–0.37]	< .01^h,i,j^
LL (cm)	5.59 [5.32–5.97]	5.77 [5.36–6.17]	6.07 [5.9–6.27]	5.59 [5.26–5.94]	< .01^c^
UmC (cm)	5.15 [4.78–5.46]	5.4 [5.17–5.65]	5.65 [5.3–5.77]	5.33 [4.69–5.5]	< .05^g,h,i^
UmA (cm^2^)	1.73 [1.55–2.05]	1.98 [1.8–2.16]	2.06 [1.93–2.24]	1.9 [1.56–2.04]	< .05^g,h,i^
UvC (cm)	2.83 [2.58–3.05]	2.94 [2.73–3.27]	2.9 [2.77–3.3]	2.9 [2.63–3.13]	.30
UvA (cm^2^)	0.57 [0.48–0.66]	0.62 [0.53–0.76]	0.62 [0.57–0.73]	0.59 [0.51–0.7]	.24
mUaC (cm)	1.42 [1.27–1.56]	1.57 [1.43–1.74]	1.57 [1.47–1.64]	1.37 [1.21–1.57]	< .01^h,i,j^
mUaA (cm^2^)	0.13 [0.11–0.16]	0.17 [0.14–0.2]	0.16 [0.15–0.19]	0.13 [0.1–0.16]	< .01^h,i^^,j^
UveA (cm^2^)	0.86 [0.67–0.97]	0.96 [0.83–1.08]	0.97 [0.89–1.14]	0.87 [0.73–1]	< .05^h,i,j^
WjA (cm^2^)	0.94 [0.76–1.06]	1.02 [0.81–1.18]	1.06 [0.97–1.27]	0.95 [0.73–1.07]	< .05^g,h,i^

Data are expressed as median [interquartile range, IQR], or as *n* (%). Continuous variables were compared using the Kruskal–Wallis rank sum test with the post-hoc Dunn's test, and for categorical variables the chi-square test with the Bonferroni correction or Fisher's exact test were applied

*GDMG1* diet-controlled gestational diabetes mellitus, *GDMG2* insulin-controlled gestational diabetes mellitus, *T1DM* type 1 diabetes mellitus, *BMI* body mass index, *HbA1c* glycated hemoglobin concentration, *BPD* biparietal diameter, *HC* head circumference, *AC* abdominal circumference, *FL* femur length, *SSFM* sub-scapular fat mass, *AFM* abdominal fat mass, *MTFM* mid-thigh fat mass, *MTLM* mid-thigh lean mass, *HeC* heart circumference, *HeA* heart area, *ThC* thorax circumference, *ThA* thorax area, *IVS* inter-ventricular septum, *LL* liver length, *UmC* umbilical cord circumference, *UmA* umbilical cord area, *UvC* umbilical vein circumference, *UvA* umbilical vein area, *mUaC* mean umbilical artery circumference, *mUaA* mean umbilical artery area, *UveA* umbilical vessels area, *WjA* Wharton's jelly area

^a^Results of the 75 g Oral Glucose Tolerance Test performed between 24 and 28 gestational weeks

^b^Fetal macrosomia defined as birth-weight over 4000 g irrespective of gestational age

^c^GDMG1, GDMG2, control vs. T1DM

^d^GDMG2 vs. T1DM, control

^e^GDMG1 vs. T1DM, control

^f^GDMG1, GDMG2 vs. control

^g^GDMG1 vs. GDMG2

^h^GDMG1 vs. T1DM

^i^T1DM vs. control

^j^GDMG2 vs. GDMG1, control

^k^GDMG2 vs. control

With regard to ultrasound measurements of the standard fetal biometric parameters, significant differences were demonstrated for the BPD and HC (Table 2). Both parameters were significantly lower in the group of T1DM patients compared to other groups (p < .05). Measurements of the fetal SSFM, AFM, MTFM, MTFM/MTLM ratio and IVS were significantly higher in patients with GDMG2/T1DM as compared to the GDMG1 and/or control groups (SSFM, AFM, IVS, p < .01; MTFM, MTFM/MTLM ratio, p < .05), and reached the highest values among patients with pre-existing diabetes (Table 2). LL proved to be an additional, statistically significant differentiation factor between T1DM and women from the other groups (p < .01) (Table 2). Furthermore, the analysis revealed that both HeC and HeA were significantly higher among patients with T1DM compared to the GDMG1 group (p < .05). Umbilical cord measurements showed a significant increase of the UmC, UmA, mUaC, mUaA, UveA and WjA in pregnancies complicated by GDMG2/T1DM as compared to the GDMG1 and/or control groups (UmC, UmA, UveA, WjA, p < .05; mUaC, mUaA, p < .01) (Table 2). Similar to the fetal biometric parameters, the UmC, UmA, UveA and WjA measurements achieved the highest values in patients with T1DM.

The separate analysis performed only in the group of macrosomic fetuses showed significant differences in relation to five parameters. The gestational age in the group of patients with T1DM was significantly lower compared to the remaining women (T1DM 38 weeks [37–38] vs. GDMG1 40 weeks [38.5–40]; GDMG2 39 weeks [38–39.5]; control 40 weeks [39–41], p < .001). Fasting glucose concentration during OGTT in GDM patients was significantly higher compared to the group of non-diabetic women (GDMG1 92.8 mg/dl [92–95.5]; GDMG2 99.5 mg/dl [98.2–100] vs. control 83 mg/dl [79–86], p < .001), and reached the highest values in patients receiving the insulin, (p < .001). Similarly, 2-h glucose concentration was significantly higher among both GDM groups as compared to controls (GDMG1 149 mg/dl [107.5–162.5]; GDMG2 155 mg/dl [130–168.7] vs. control 104 mg/dl [95.5–114], p < .001). With regard to the fetal biometric parameters, the only significant differences were found for the BPD and HC measurements. Both parameters were significantly lower in women with T1DM-complicated pregnancy compared to the GDMG1 and control groups (BPD T1DM 9.26 cm [8.86–9.45] vs. GDMG1 9.58 cm [9.33–9.7]; control 9.54 cm [9.41–9.68], p < .05), (HC T1DM 32.9 cm [32.5–34.1] vs. GDMG1 35.1 cm [34.3–35.7]; control 35 cm [34.4–35.3], p < .05).

**Fig. 2:**
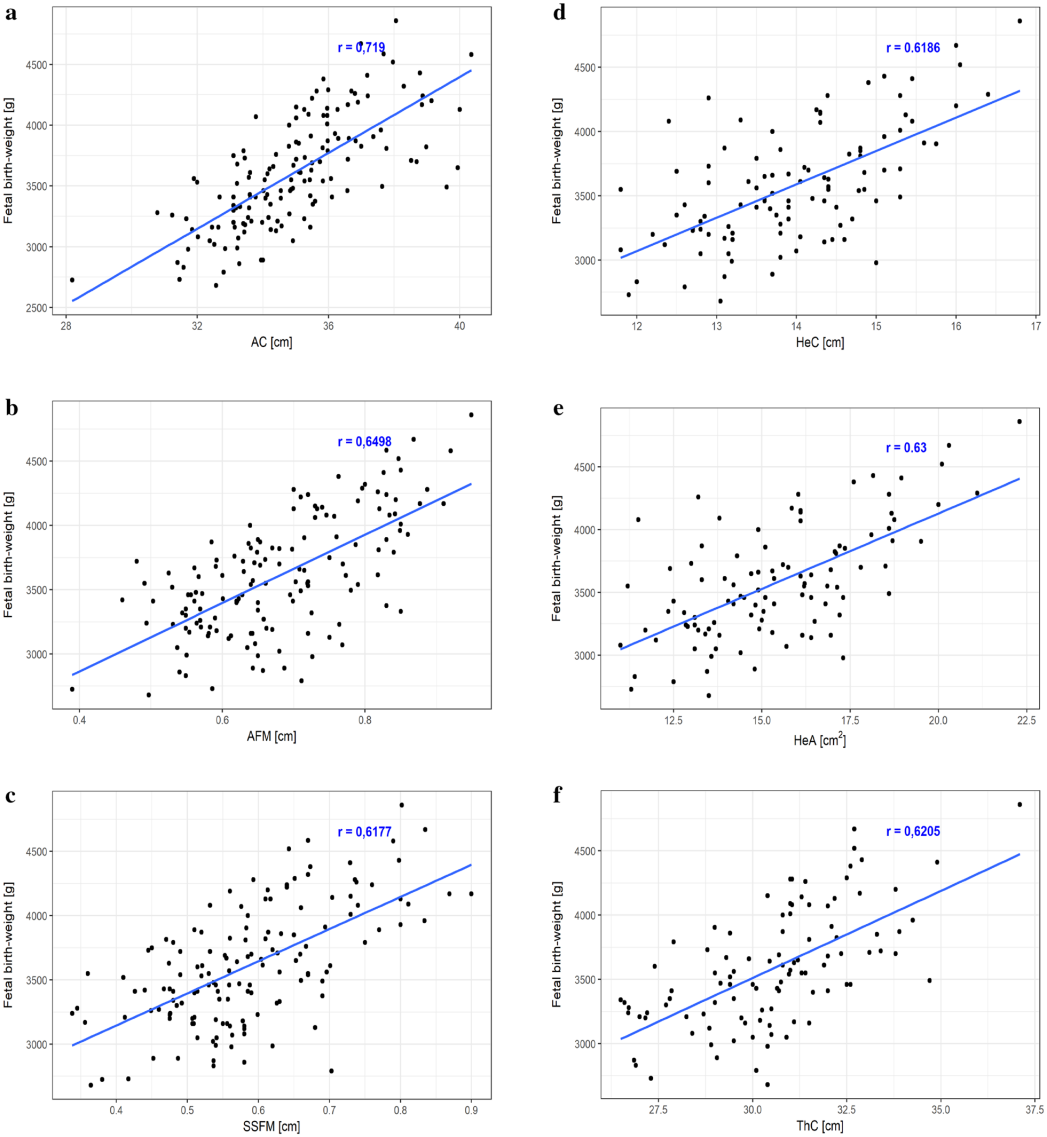

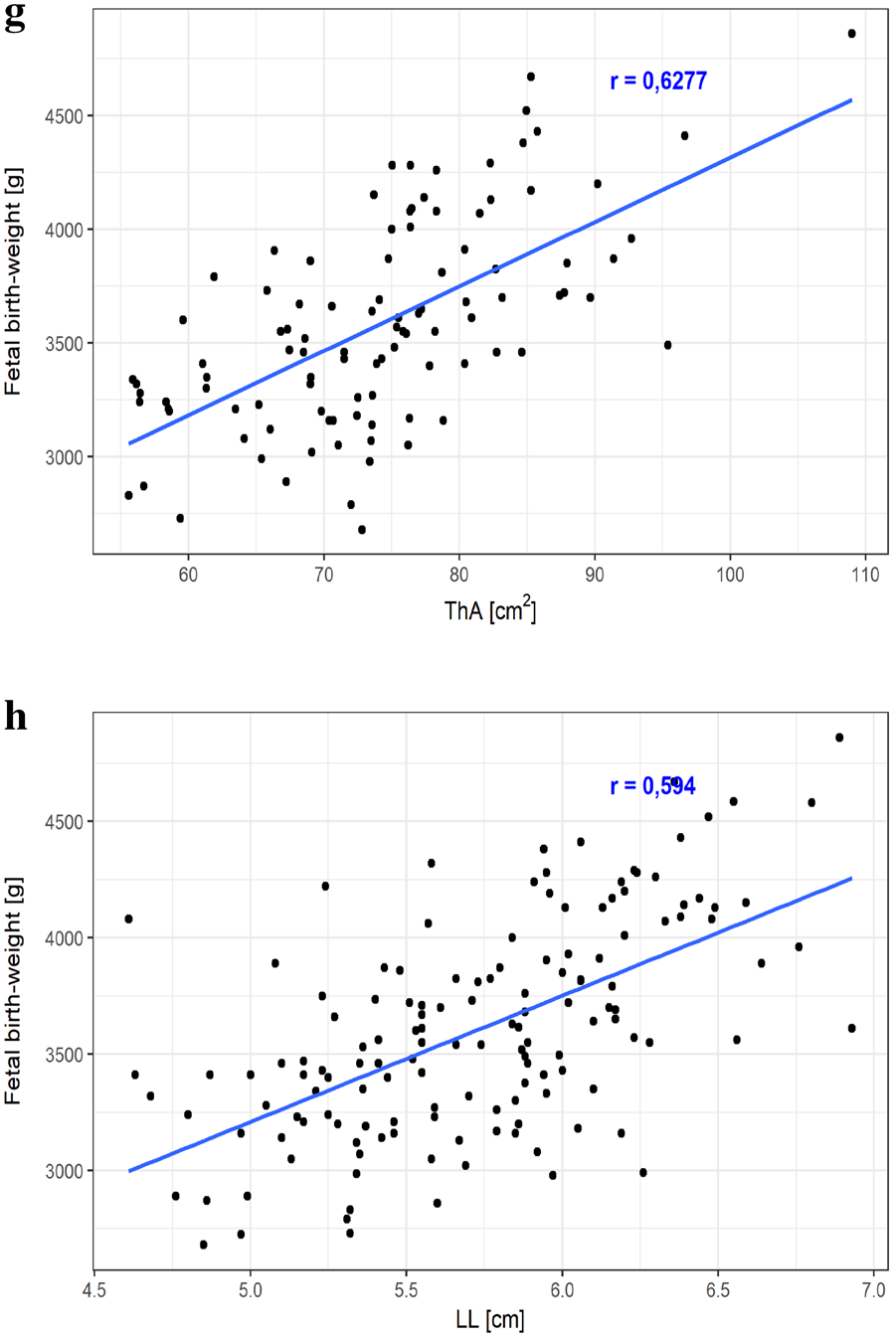
Correlations between the fetal birth-weight and selected maternal–fetal parameters (**a**–**h**). *AC* abdominal circumference, *AFM* abdominal fat mass, *SSFM* sub-scapular fat mass, *HeC* heart circumference, *HeA* heart area, *ThC* thorax circumference, *ThA* thorax area, *LL* liver length. All results expressed as Pearson's correlation coefficient (r), p < .001

The correlation analysis performed in the total study population demonstrated presence of strong, positive correlations between the FBW and the AC (r = 0.72, p < .001), AFM (r = 0.65, p < .001), SSFM (r = 0.62, p < .001), HeC (r = 0.62, p < .001), HeA (r = 0.63, p < .001), ThC (r = 0.62, p < .001), ThA (r = 0.63, p < .001) and LL (r = 0.59, p < .001) ultrasound measurements (Fig. 2a–h). Additionally, positive correlations were found between the FBW and maternal pre-pregnancy weight (r = 0.17, p < .05), gestational weight gain (r = 0.16, p < .05), maternal height (r = 0.31, p < .001), 3rd trimester HbA1c concentration (r = 0.48, p < .001), BPD (r = 0.38, p < .001), HC (r = 0.42, p < .001), FL (r = 0.44, p < .001), IVS (r = 0.47, p < .001), MTFM (r = 0.40, p < .001), MTLM (r = 0.19, p < .05), MTFM/MTLM ratio (r = 0.32, p < .001), UmC (r = 0.30, p < .001), UmA (r = 0.27, p = .001), UvC (r = 0.19, p < .05), UvA (r = 0.24, p < .01), UveA (r = 0.26, p < .01) and WjA (r = 0.19, p < .05).

The results of multivariate linear regression analysis demonstrating independent contribution of maternal–fetal parameters to the FBW are shown in Table 3. Among the variables included in the final model maternal height and ultrasound measurements of the fetal BPD, AC, AFM and LL were the strongest predictors of the fetal birth-weight in the total study population. The constructed logistic model revealed that an increase in the ultrasound measurements of the fetal AFM (mm) [OR 3.98, 95% CI (2.0–8.70), p < .001], AC (cm) [OR 1.37, 95% CI (1.0–1.9), p < .05], and FL (mm) [OR 1.23, 95% CI (1.0–1.5), p < .05] was associated with a significant risk of fetal macrosomia > 4000 g occurrence. There were no differences in the parameters selected by the logistic model between the diabetic and control populations.

**Table 3:** Results of the multivariate linear regression analysis: factors contributing to the fetal birth-weight (adjusted R^2^ = .69)

	Estimate	95% CI	*p* value
Gestational age (week)	45.0	− 5.1 to 95.1	.08
Maternal height (cm)	12.8	5.0–20.5	< .01
BPD (mm)	14.7	0.6–28.9	< .05
AC (cm)	84.2	57.7–110.8	< .001
FL (mm)	11.3	− 0.5 to 23.2	.06
AFM (mm)*	101.4	43.8–158.9	< .001
LL (mm)	14.8	3.6–25.9	< .05
T1DM	124.7	− 37.1 to 286.6	.13

*AC* abdominal circumference, *AFM* abdominal fat mass, *BPD* biparietal diameter, *FL* femur length, *LL* liver length, *T1DM* type 1 diabetes mellitus, *CI* confidence interval

*AFM showed strong interdependence with the sub-scapular fat mass (SSFM) measurement, therefore, for the linear regression analysis only AFM was selected. Linear model including SSFM revealed that it was also an independent predictor of the fetal birth-weight [63.0, 95% CI (1.4–124.7), p < .05; adjusted R^2^ = .66]

**Fig. 3:**
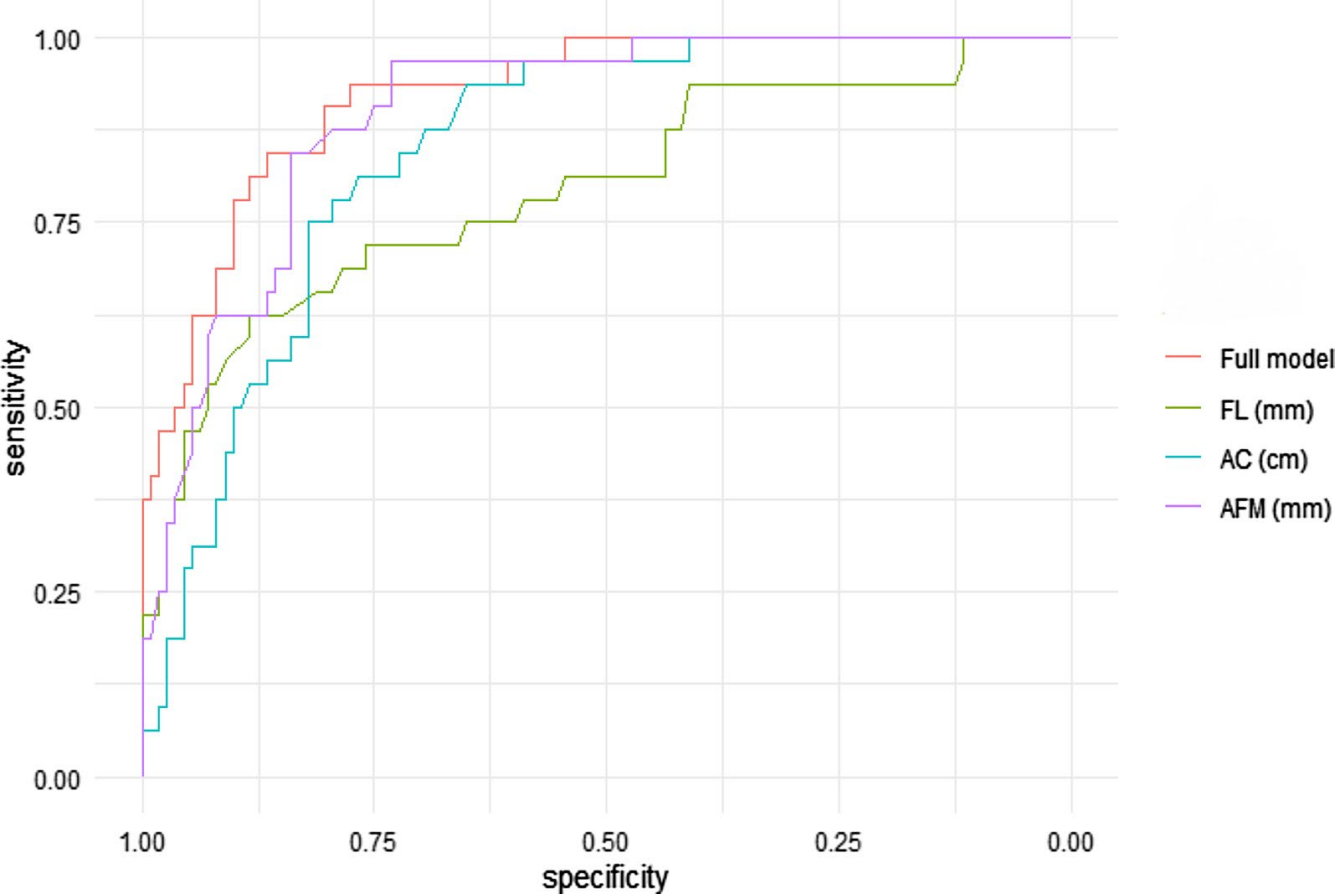
ROC curves evaluating the performance of AC, AFM and FL measurements in the prediction of fetal macrosomia. *AC* abdominal circumference, *AFM* abdominal fat mass, *FL* femur length

The results of a ROC analysis for separate AC, AFM, and FL measurements as well as the full model incorporating all three variables are shown in Fig. 3. The analysis revealed the highest AUC for the full model (0.923) with the sensitivity of 93.8%, specificity 77.7%, PPV 54.5% and NPV 97.8% for the prediction of fetal macrosomia (Table 4). The equation used for the estimation of risk of the delivery of a fetus weighing > 4000 g is given below:

$$\log \left( {\frac{p}{1 - p}} \right) = - 37,994 + 0.205*{\text{FL }}\left( {{\text{mm}}} \right) + 0.316*{\text{AC }}\left( {{\text{cm}}} \right) + 1.382*{\text{AFM }}\left( {{\text{mm}}} \right)$$

**Table 4:** Diagnostic performance of AC, AFM and FL measurements in prediction of fetal macrosomia

	FL (mm)	AC (cm)	AFM (mm)	Full model
Threshold (p)	0.337	0.159	0.144	0.138
Cut-off value	77.5	34.97	6.99	–
AUC (95% CI)	0.794(0.696 –0.892)	0.850 (0.785–0.914)	0.900 (0.848–0.952)	0.923 (0.877–0.970)
Sensitivity (%)	62.5	93.8	96.9	93.8
Specificity (%)	88.4	65.2	73.2	77.7
PPV (%)	60.6	43.5	50.8	54.5
NPV (%)	89.2	97.3	98.8	97.8

*AC* abdominal circumference, *AFM* abdominal fat mass, *FL* femur length, *AUC* area under the curve, *CI* confidence interval, *PPV* positive predictive value, *NPV* negative predictive value

The p in the left-side of equation is the threshold of the full model (0.138), therefore, results of the right-side of equation ≥ − 1.832 are predictive for fetal macrosomia.

Using a linear regression analysis, a new formula incorporating the variables selected in the logistic model (AC, AFM, FL) was constructed to estimate birth-weight of the fetus (EFW):

$$EFW\left( g \right) = - 2254,942 + 17,204*{\text{FL }}\left( {{\text{mm}}} \right) + 105,531*{\text{AC }}\left( {{\text{cm}}} \right) + 131,347*{\text{AFM }}\left( {{\text{mm}}} \right)$$

Table 5 presents the mean absolute percent errors and frequency of predictions within 10% of error for the new (AC, AFM, FL) and the Hadlock (BPD, HC, AC, FL) formulas. Although there was no significant difference with respect to the mean absolute percent error between the two formulas in the total study population (6.4% vs. 7.2%, p = .09), the new formula provided significantly lower error in the sub-group of patients with T1DM (5.7% vs. 9.4%, p < .05). In addition, using the new formula, the percentage of estimations within 10% of error was significantly higher in the total study population (78.5% vs. 72.2%, p < .001) as well as among the patients with GDMG1 (85.0% vs. 77.5%, p < .01) and controls (82.5% vs. 72.5%, p < .001).

**Table 5:** Comparison of accuracy of the new and the Hadlock formulas [36] to estimate birth-weight of the fetus

	GDMG1 (n = 40)	GDMG2(n = 40)	T1DM (n = 24)	Control (n = 40)	Total (n = 144)
New formula (AC, AFM, FL)					
APE (%)^a^	5.8 ± 3.7	7.8 ± 5.6	5.7 ± 4.1*	6.1 ± 4.3	6.4 ± 4.6
Estimations within 10% of APE	85.0% (34/40)**	67.5% (27/40)	79.2% (19/24)	82.5% (33/40)***	78.5% (113/144) ***
Hadlock formula (BPD, HC, AC, FL)					
APE (%)^a^	6.4 ± 5.5	6.8 ± 5.3	9.4 ± 6.7	6.9 ± 4.5	7.2 ± 5.4
Estimations within 10% of APE	77.5% (31/40)	72.5% (29/40)	62.5% (15/24)	72.5% (29/40)	72.2% (104/144)

Data are expressed as mean ± SD, or as %

*GDMG1* diet-controlled gestational diabetes mellitus, *GDMG2* insulin-controlled gestational diabetes mellitus, *T1DM* type 1 diabetes mellitus, *BPD* biparietal diameter, *HC* head circumference, *AC* abdominal circumference, *FL* femur length, *AFM* abdominal fat mass

^a^APE-absolute percent error = (estimated fetal birth-weight − fetal birth-weight) * 100/fetal birth-weight

*p < .05; **p < .01; *** p< .001 vs. Hadlock formula

## Discussion

In the presented study, we evaluated the non-standard biometric parameters of fetuses in pregnancies complicated by GDM/T1DM. The fetal soft-tissue (SSFM, AFM, MTFM), heart circumference/area, IVS thickness, LL as well as certain umbilical cord (UmC/UmA, UaC/UaA, UveA, WjA) measurements constituted the group of parameters significantly increased in the population of mothers with diabetes. Importantly, the observed increase was most prominent among women with diabetes requiring therapy with insulin (GDMG2/T1DM).

The increase of SSFM, AFM, and MTFM measurements among fetuses and neonates from pregnancies complicated by GDMG2 or T1DM is consistent with the observations of other authors [9, 12, 33]. Longitudinal studies performed in a population of women with different degrees of glucose intolerance including GDM and T1DM demonstrated a significant and progressive increase of the abdominal, sub-scapular and mid-thigh fetal adiposity as early as 25–26 gestational weeks as well as a positive correlation of measurements with the FBW [19, 20, 24, 29, 33, 37, 38]. Interestingly, similar to previous publications, increased fat deposition in fetuses from GDMG2/T1DM groups was observed despite optimal glycemic control in the third trimester of pregnancy [33]. As HbA1c reflects only average glycemia, regardless of glucose fluctuations and hyperglycemic excursions, it is plausible that in patients with more severe glucose homeostasis impairment (GDMG2/T1DM), increased transplacental flux of energy substrates to the fetus is present throughout gestation even under strict glycemia management [39].

In primates, cardio- and hepatomegaly are the most common pathologies found in fetuses under the conditions of chronic hyperinsulinemia [5]. Likewise, an increase in the LL and IVS measurements was noted in the fetuses of diabetic mothers [21, 22, 25, 26, 40–42]. Regarding the liver, a significant increase in length becomes evident as early as the 18th week of pregnancy, and marked progress towards the end of gestation is observed in women with pre-existing diabetes [40]. Furthermore, increased LL measurements are already observed between 21 and 24 gestational weeks in patients who develop GDM, and a positive correlation with fasting glucose concentration during OGTT was noted [21, 22]. With regard to cardiac morphological parameters, in the majority of studies, increase in the IVS thickness was the only significant difference between fetuses from normoglycemic and GDM/T1DM-complicated pregnancies [25, 26, 41, 42]. Importantly, despite the increase in the thickness of the IVS, in well-controlled diabetes, it was classified as a pathological hypertrophy in 20–60% of cases, and the only accompanying hemodynamic effects were a lower early/late diastolic peak flow velocity index of the tricuspid valve in conjunction with a higher aortic or pulmonary artery peak systolic velocity [25, 26, 41, 42]. In our diabetic population, a significant increase in the LL and IVS measurements was observed among women receiving insulin (GDMG2/T1DM) during the course of pregnancy. Although in the majority of women from both groups, glycemic control was assessed as optimal (HbA1c ≤ 6%), the increase of the LL and IVS measurements corresponded with significantly higher HbA1c concentrations in the 3rd trimester of gestation as compared to the GDMG1 and control patients.

Our observations on increased umbilical cord measurements in diabetic pregnancies and their correlation with the FBW are similar to previous publications [27, 35, 43]. Nonetheless, significant differences were observed only in the group of patients requiring insulin treatment (GDMG2/T1DM), and the increase in parameters such as UmA was mainly due to the increase in the measurements of the Wharton’s jelly and umbilical arteries areas. The above observations may be explained by the fact that a high concentration of growth factors such as IGF-1, transforming growth factor β (TGF-β), or fibroblast growth factor (FGF) were found within both tissue compartments of the umbilical cord [11]. As a result, in pregnancies complicated with GDMG2/T1DM, characterized by high concentrations of insulin and leptin in the cord blood, stimulation of Wharton’s jelly cells and to a lesser extent of arterial walls may occur, which ultimately leads to the increased production of the extracellular matrix proteins [6]. Despite a positive correlation between the parameters of the umbilical cord and the FBW, neither of them turned out to be an independent predictor of the FBW or fetal macrosomia. In accordance with our findings, in a study by Cromi et al., measurement of the cord area > 95th percentile allowed for the detection of only 25% of newborns weighing more than 4000 g [35].

In the available literature parameters such as gestational age, fasting glucose concentration, maternal height, pre-pregnancy weight, and gestational weight gain proved to be the main factors determining the FBW in GDM and non-diabetic populations [12]. Among the above-mentioned parameters, in our analysis including multiple maternal–fetal biometric data, only the mother’s height constituted an independent predictor of the FBW. Although the gestational age entered the regression model, it did not reach the level of significance, and both the pre-pregnancy weight and gestational weight gain demonstrated only a weak correlation with the FBW. The lack of a proven effect of the fasting glucose concentration may be due to the limited number of patients with GDM in the presented study. However, it should be noted that in the group of women with GDM treated with insulin, significantly higher fasting glucose levels during OGTT were recorded in conjunction with increased FBW and fat mass thickness as compared to the GDMG1 and control patients. Similarly, in a study by Uvena-Celebrezze et al., fasting glucose showed the strongest correlation with the neonatal fat mass, sum of skinfold thicknesses, and FBW in GDM-complicated pregnancies [44]. It should also be mentioned that in the presented study, the 3rd trimester HbA1c concentration, which is a determinant of long-term glycemia control, was significantly higher among women with GDMG2/T1DM compared to the GDMG1 group, and remained in moderate correlation with the FBW.

The comparative analysis between macrosomic fetuses from diabetic and normoglycemic pregnancies did not reveal significant differences in relation to any of the non-standard biometric parameters. On the contrary, in some of the previous studies, measurements of parameters such as WjA were significantly higher among the macrosomic fetuses of diabetic mothers [35]. Regarding the maternal data, fasting and 2-h glucose concentrations during OGTT were significantly higher among GDM women who delivered fetuses weighing > 4000 g, in particular when therapy with insulin was administered. The above observation once again highlights the importance of fasting glucose concentration as a predictive factor in relation to the severity of glucose intolerance, and the occurrence of fetal macrosomia in the population of women diagnosed with GDM [12].

Fetal macrosomia > 4000 g constitutes frequent complication in pregnancies with concomitant GDM/T1DM and is associated with numerous adverse perinatal outcomes including prolonged labor, cephalopelvic dysproportion, shoulder dystocia, increased risk of Cesarean section, birth injuries, post-partum hemorrhage, low Apgar scores as well as increased neonatal mortality [45, 46]. As a consequence preventive measures aimed at the most accurate estimation of the FBW are of particular importance for clinicians deciding on the mode of delivery. Regrettably, studies conducted so far demonstrated that in the case of fetal macrosomia different ultrasound formulas tend to underestimate FBW by at least 300 g with the sensitivity and specificity of calculations in the diabetic population varying between 33–69% and 77–98%, respectively [47, 48]. Furthermore, it was noted that ultrasound scans performed within 7 days from delivery underestimate FBW by more than 15% in 26.3% of diabetes-complicated pregnancies as compared to 5.4% of normoglycemic gestations [49]. According to a widely accepted opinion one of the main factors responsible for the observed measurement errors in ultrasound examination, in particular among diabetic patients is the fact that fetus has an irregular three-dimensional body of varying density and tissue composition.

In the study by Jazayeri et al. AC measurement of ≥ 35 cm performed within the 2 week period before delivery exhibited the highest correlation coefficient with the FBW (0.95) and PPV for fetal macrosomia of almost 93% [50]. Similarly, in our population consisting of women diagnosed with GDM/T1DM AC showed the strongest correlation with the FBW (0.72). On the other hand AFM measurement performed better with respect to sensitivity, specificity and AUC values when predicting fetal macrosomia. Studies conducted so far in diabetic population failed to provide unambiguous explanation of such observations. In the study by Garabedian et al. sensitivity and AUC of AFM were higher than AC, results of Higgins et al. demonstrated higher sensitivity but lower AUC and positive likelihood ratio values of the former, finally Bethune et al. revealed higher specificity and positive likelihood ratio of the abdominal fat measurement [18, 29, 30]. Differences in the characteristics of study population (GDM and/or T1DM), week of gestation in which ultrasound was performed in conjunction with various definitions of fetal macrosomia (i.e. EFW ≥ 90 percentile for gestational age) constitute possible explanations of the above-mentioned discrepancies.

In the study by Scioscia et al. evaluating 35 different ultrasound formulas, those including AC and FL measurements showed the highest accuracy for newborns weighing more than 4000 g [47]. Similarly, our logistic model revealed that the risk of fetal macrosomia increased markedly following an increase in the AFM, AC, and FL measurements. The relationship between the above-mentioned biometric parameters and fetal overgrowth in GDM/T1DM-complicated pregnancies is well-established in the literature [18, 23, 24, 29, 30]. Our equation constructed on the basis of a logistic model to estimate the risk of fetal macrosomia was characterized by high accuracy (AUC = 0.923), sensitivity (93.8%) and NPV (97.8%). On the other hand, the high rate of false positive results, and thus low PPV (54.5%) may raise certain concerns. Importantly, the formula using the same parameters to estimate fetal birth-weight in the antenatal period allowed for a significant reduction in the mean absolute percent error among T1DM women exposed to the highest risk of fetal macrosomia. In 2003, Bethune et al. suggested that the combined use of AFM and AC measurements may improve the prediction of fetal macrosomia in pregnancies complicated by GDM [18]. Our and other authors’ observations confirmed this assumption in populations consisting of GDM/T1DM patients, and AFM, due to the ease of measurement and high reproducibility, is worthy of further evaluation [23, 24, 29, 30].

Apart from the relatively small number of patients with T1DM, the present study is limited by the lack of data concerning glycemic control in the first and second trimesters of pregnancy. The latter may be of importance given that high HbA1c levels in early pregnancy are a strong predictor of accelerated fetal growth and macrosomia [51]. We also acknowledge the fact that our population consisting of diabetic and healthy patients is somewhat "artificial" and confined to Caucasian women in singleton pregnancy with normal amniotic fluid index. As a consequence of such selection of study participants performance of developed equations may differ depending on the heterogeneity of population. Nonetheless, it is the first study to evaluate the relationships between multiple biometric parameters of the fetus and the type of diabetes, FBW, and macrosomia occurrence in well-defined groups of GDM/T1DM patients. Moreover, two new formulas incorporating fetal adiposity parameter (AFM) may allow for a better estimation of the FBW and risk of fetal macrosomia in diabetes-complicated pregnancies.

## Conclusions

Summing up, the study results demonstrated a significant increase of the fetal soft tissues, IVS, LL, and umbilical cord ultrasound-derived measurements in pregnancies with concomitant GDMG2/T1DM. In addition to standard biometric measurements, some of the parameters, such as AFM, may find application in the monitoring of fetal growth, estimation of the FBW and prediction of fetal macrosomia. As a result proposed formulas incorporating AFM measurement may help in the management of diabetes-complicated pregnancies by allowing clinicians to decide on the mode of delivery more adequately, and thus decrease the rate of maternal–fetal complications associated with fetal overgrowth. Prospective large-cohort studies in more heterogeneous populations of women need to be performed for further validation of AFM clinical applicability.

## Data Availability

The data that support the findings of this study are available from the corresponding author on reasonable request. The data are not publicly available due to privacy or ethical restrictions.

## Abbreviations

AC: Abdominal circumference
AFM: Abdominal fat mass
BPD: Biparietal diameter
FL: Femur length
GDM: Gestational diabetes mellitus
HC: Head circumference
HeA: Heart area
HeC: Heart circumference
IVS: Inter-ventricular septum
LL: Liver length
MTFM: Mid-thigh fat mass
MTLM: Mid-thigh lean mass
SSFM: Sub-scapular fat mass
T1DM: Type 1 diabetes mellitus
ThA: Thorax area
ThC: Thorax circumference
UaA: Umbilical artery area
UaC: Umbilical artery circumference
UmA: Umbilical cord area
UmC: Umbilical cord circumference
UvA: Umbilical vein area
UvC: Umbilical vein circumference
UveA: Umbilical vessels area
WjA: Wharton's jelly area
